# A Real-World Experience With Resmetirom: Tolerability and Access

**DOI:** 10.1016/j.gastha.2025.100709

**Published:** 2025-05-20

**Authors:** Sameera Shuaibi, Ian Tobal, James Gore, Ruona Ebiai, Claire Ozoral, George Therapondos

**Affiliations:** 1 Department of Internal Medicine, Ochsner Health, New Orleans, Louisiana; 2 Specialty Pharmacy, Ochsner Health, New Orleans, Louisiana; 3 Multiorgan Transplant, Ochsner Health, New Orleans, Louisiana and University of Queensland, Brisbane, Australia

**Keywords:** Insurance Obstacles, Medication Complications, Prescription Disparities

## Abstract

**Background and Aims:**

Metabolic dysfunction–associated steatohepatitis (MASH) is a progressive liver disease often leading to cirrhosis. Resmetirom is the first Food and Drug Administration–approved treatment for MASH. The aim of this study was to evaluate the tolerability of resmetirom for the treatment of MASH fibrosis and factors affecting access, including insurance coverage and dispensing delays for patients that received at least 3 months of therapy.

**Methods:**

A retrospective chart review conducted at a large tertiary hospital for all (113) MASH patients prescribed resmetirom between April 2024 to September 2024 and who were treated for at least 3 months to assess their tolerability and any obstacles medication access.

**Results:**

Resmetirom was prescribed for 137 patients, of whom 113 (85.5%) completed at least 3 months of treatment and met the inclusion criteria. Pertinent adverse effects were nausea and vomiting (12.4%) and diarrhea (12.4%). The mean baseline aspartate aminotransferase, alanine aminotransferase, and alkaline phosphatase values were 40.15 international units per liter (IU/L), 49.96 IU/L, and 83.04 IU/L, respectively. At 3 months of treatment, mean aspartate aminotransferase, alanine aminotransferase, and alkaline phosphatase values decreased to 36.15 IU/L, 45.38 IU/L, and 81.33 IU/L, respectively. Regarding insurance coverage for all patients prescribed resmetirom, 33 patients (24.1%) required an appeal, with 93.4% (128) ultimately receiving insurance approval. Average delay in prescription fill after insurance approval was 12.5 days. Resmetirom discontinuation incurred among 10 (8.8%) patients.

**Conclusion:**

Resmetirom is a safe medication with good tolerability in regard to the side effect profile for the first 3 months. Most patients were able to gain insurance approval but delays in dispensing and insurance requirements of invasive fibrosis testing to obtain approval were observed.

## Introduction

Metabolic dysfunction–associated steatohepatitis (MASH) is one of the leading causes of chronic liver disease worldwide.^1^ The global prevalence of metabolic dysfunction–associated steatotic liver disease (MASLD) is approximately 31%.^2^ Management has until recently been lifestyle modification and, in some cases, bariatric surgery to halt the progression of the disease in the absence of effective pharmacological therapies. Disease progression to advanced fibrosis/cirrhosis and subsequent decompensation allows limited treatment options such as liver transplantation.^3^ As such, early recognition and treatment of disease is crucial. In March 2024, resmetirom (Rezdiffra TM) was approved by the United States Food and Drug Administration (FDA) to treat noncirrhotic MASH with moderate to advanced liver fibrosis (stages F2 and F3).^4^ Resmetirom is available in three oral doses 60 mg, 80 mg, and 100 mg to be taken once daily. It is a liver-directed, thyroid hormone receptor beta-selective agonist that improves lipid metabolism, glucose homeostasis, and inflammation.^5^ It has shown promising improvements in MASH histology amongst those who were treated with it in its current Phase 3 trials.

## Aims

This medication is currently mainly prescribed in specialized liver clinics, and we wanted to assess real-world utilization of this medication at our tertiary center. We evaluated how fibrosis was assessed and how patients were deemed appropriate for the treatment and collected patient characteristics. We also evaluated whether patients were able to get access to the medication through their insurance, and we investigated insurance denials, delays in dispensation and factors that led to premature discontinuation. Additionally, we also wanted to detail the medication’s tolerability, and any unfavorable outcomes experienced. This study collected data for the first 3 months of treatment.

## Methods

### Study Design

This retrospective observational descriptive study was conducted at Ochsner Medical Center, a large tertiary hospital, to evaluate patients prescribed resmetirom between April 2024 and September 2024. Patient data were identified using Slicer Dicer and log records from the Ochsner specialty pharmacy. Initially, 137 patients met the inclusion and exclusion criteria. Patient data were collected at baseline (before initiating resmetirom) and at 1 month and 3 months. No deaths occurred during the study.

Patient groups were divided into those who received 60 mg, 80 mg, or 100 mg of resmetirom in a once daily fashion. Dosing was decided according to the weight-based prescription guidelines. Eligible patients had F2 or F3 fibrosis confirmed via FibroScan (vibration-controlled transient elastography) or magnetic resonance elastography (MRE). Baseline characteristics recorded included age, gender, ethnicity, body mass index (BMI), comorbidities, and concurrent medications. In addition to clinical outcomes, prescription and insurance-related data were collected for all patients prescribed resmetirom, including prescriber specialty and credentials, initial dose prescribed, insurance/payer type, prior authorization requirements, financial assistance status, approval timelines, and dispensing pharmacy. Patients were monitored throughout the study period for primary and secondary endpoints to assess treatment response and access barriers.

### Data Collected

Tolerability and side effects associated with resmetirom in patients with MASLD treated in routine clinical settings were noted. Side effects included were as follows: nausea, vomiting, diarrhea, dizziness, rash/pruritus, abdominal pain, cholecystitis, pancreatitis, hepatotoxicity.

Changes in alanine aminotransferase (ALT), aspartate aminotransferase (AST), alkaline phosphatase (ALP) at 1 and 3 months were also collected. Other collected data were hospitalizations, dose of resmetirom used, changes of resmetirom dose, resmetirom discontinuation and changes to nonresmetirom daily medication doses while patients were on resmetirom. We also assessed patient BMI and weight at 1 and 3 months, any interactions with other prescribed medications such as statins, clopidogrel, glucagon-like peptide-1 agonist. Data on comorbidities such as diabetes, hypertension, and hyperlipidemia were also noted.

#### Inclusion criteria

• Adults aged 18 or older
• Confirmed diagnosis of MASLD based on liver biopsy or imaging (fibroscan or MRE)
• Treatment with resmetirom for at least 3 months
• Availability of clinical follow-up data for at least 3 months

#### Exclusion criteria

• Patients with significant alcohol use (>20 g/day for women, >30 g/day for men)
• Other causes of liver disease (eg, viral hepatitis, autoimmune hepatitis)
• Incomplete medical records or lack of follow-up data

### Statistical Analysis

Descriptive data were developed from patient data collection.

### Ethical Approval

Only institutional review board–approved research team members who have current Health Insurance Portability and Accountability Act, Good Clinical Practice, and human subjects' protection training were authorized to access patient records, and the protocol was approved by the Ochsner IRB, ID #2024.323 on October 30, 2024.

## Results

### Clinical Results

#### Medication initiation

Of the 137 patients prescribed resmetirom, 113 (82.5%) began taking the medication and were included in this portion of the study.

#### Demographics and comorbidities

The mean age of this group at treatment start was 55.58 years with ages ranging from 21 to 74 years. Forty-six (40.7%) were male and 67 (59.3%) were female. Ninety-four (83.2%) patients identified as Caucasian, 9 (8%) African American, 7 (6.1%) as Hispanic, 1 (0.9%) as Asian, and 2 (1.8%) as other (Table 1). Of this study population at the time of treatment initiation, the mean BMI was 35.66 kg/m^2^ (range: 23.4–60.8 kg/m^2^). 62 (54.9%) had diabetes mellitus, 73 (64.6%) had hypertension, 82 (72.6%) had hyperlipidemia, 18 (15.9%) were overweight (BMI >25), 89 (78.8%) were obese (BMI>30), 28 (24.8%) had obstructive sleep apnea, 11 (9.7%) had coronary artery disease, and 17 (15%) had chronic kidney disease. Regarding other medications being taken by the study population, 58 (51.3%) were taking glucagon-like peptide-1 receptor agonists, 54 (47.8%) were taking statins, and 1 (0.9%) was taking a P2Y12 inhibitor; 7 (6.2%) patients required other medication adjustments at treatment initiation 6 (5.3%) patients had their statin decreased and 1 (0.9%) had their fibrate discontinued.

**Table 1:** Demographics

Mean age (y)	Gender (n = 113)	Race (n = 113)
55.58	Male: 46 (40.71%)	Caucasian: 94 (83.20%)
	Female: 67 (59.30%)	African American: 9 (7.96%)
		Hispanic: 7 (6.19%)
		Asian: 1 (0.88%)
		Other: 2 (1.77%)

#### Fibrosis assessment

Baseline fibrosis 4 index and vibration-controlled transient elastography kPa scores were calculated on patients with available data. Fib4 score was able to be calculated on 110 patients, with an average Fib4 score of 1.44. The mean fibrosan kPa score was 10.58 and 3.69 via MRE. Their baseline fibrosis staging was determined by biopsy, MRE, Fibroscan, and/or fibrosis 4 index. Patients who underwent fibroscan amount to (89) 78%, MRE (22) 21.23%, and liver biopsy (42) 37.17%. The commonest detected stage on fibroscan and biopsy was the F3 fibrotic stage equaling (41) 23% on fibroscan and (26) 23% of liver biposy. Even though the FDA approved the medication for F2 and F3 stages of fibrosis, in real life it was witnessed that 2.65% ^3^ of the patients were found to pertain to F1 stage on fibroscan and similarly 1 patient who was found with F1 stage on liver biopsy. Nonetheless, F1 staging of fibrosis was the least predominant amongst all modalities. Mean baseline AST, ALT, and ALP were 40.15 international units per liter (IU/L) (range: 6–115 IU/L), 49.96 IU/L (range: 8–160 IU/L), and 83.04 IU/L (range: 15–155 IU/L), respectively. Table 2 and 3.

**Table 2:** Baseline Characteristics

Patient comorbidities (n = 113)		Number of patients taking a medication that can interact with resmetirom	Patient baseline liver fibrosis scores
Hyperlipidemia: 82 (72.57%)	Obese: 89 (78.76%)	Glucagon-like peptide agonists: 58 (51.33%)	F1: 0 (0%)
Hypertension: 73 (64.60%)	OSA: 28 (24.78%)	Statins: 54 (47.79%)	F2: 46 (40.71%)
Diabetes: 62 (54.87%)	Coronary artery disease: 11 (9.73%)	P2Y12 inhibitors: 1 (0.88%)	F3: 66 (58.41%)
Overweight: 18 (15.93%)	CKD: 17 (15.04%)		F4: 1 (0.88%)

CKD, chronic kidney disease; OSA, obstructive sleep apnea.

**Table 3:** Staging of Fibrosis per Modality

	Fibroscan n (%)	Liver biopsy n (%)	MRE n (%)
Number of patients who underwent the modality	89 (78.76%)	42 (37.17%)	22 (21.23%)
F1 stage fibrosis	3 (2.65%)	1 (0.88%)	0 (0%)
F2 stage fibrosis	37 (32.74%)	15 (13.27%)	20 (17.70%)
F3 stage fibrosis	41 (36.28%)	26 (23.01%)	1 (0.88%)
F4 stage fibrosis	7 (6.19%)	1 (0.88%)	2 (1.77%)
Mean kPa	10.58		3.69

#### Tolerability

**Figure 1:**
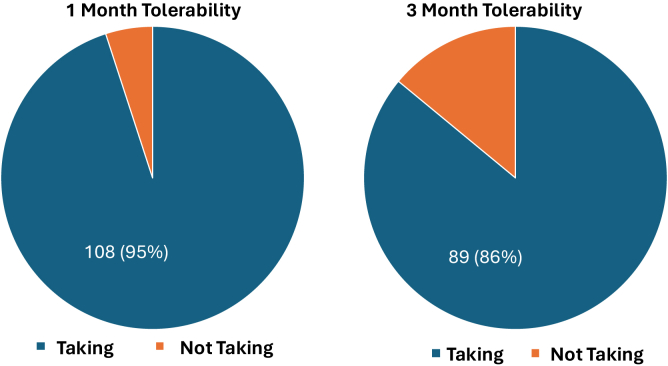
Tolerability of the medication at 1 month and 3 months. At 1 month (left) 95% of the patients appeared to be taking it whereas 86% of the patients were still taking it at the 3 month mark (right).

At the 1-month treatment mark, 108 of 113 (95%) patients were still taking resmetirom. At the 3-month treatment mark, 89 of 103 (86%) patients were still taking the medication (Figure 1). Ten (8.8%) patients had their resmetirom discontinued, 4 (3.5%) reported no longer taking it, and 10 (8.8%) patients were lost to follow-up at 3 months. Of those who discontinued resmetirom, 3 (2.63%) saw improvements in their weight and decided to discontinue pharmacotherapy and take up lifestyle modifications instead, 6 (5.26%) due to side effects of nausea and diarrhea, and 1 (0.88%) due to derangements in liver function tests (LFTs). Eight (7%) individuals had their dose of the treatment medication reduced mostly due to worsening of the nausea, vomiting, or their diarrhea.

#### Adverse events

Patients reported the following: nausea and vomiting: 14 (12.4%), diarrhea: 14 (12.4%), abdominal pain: 6 (5.3%), rash or pruritus: 4 (3.5%), and hepatotoxicity: 1 (0.9%). In regards to hepatotoxicity, elevated LFTs were witnessed in 1 patient at month 1 but decreased again by the third month mark. There were no reports of dizziness, cholecystitis, or pancreatitis during the first 3 months of treatment. No deaths occurred due to the usage of this medication Table 4.

**Table 4:** Adverse Events Reported

Adverse events reported (n = 113)	
Nausea/Vomiting: 14 (12.39%)	Dizziness: 0 (0%)
Diarrhea: 14 (12.39%)	Cholecystitis: 0 (0%)
Abdominal pain: 6 (5.31%)	Pancreatitis: 0 (0%)
Rash/Pruritus: 4 (3.54%)	Deaths: 0 (0%)
Hepatotoxicity: 1 (0.88%)	

**Figure 2:**
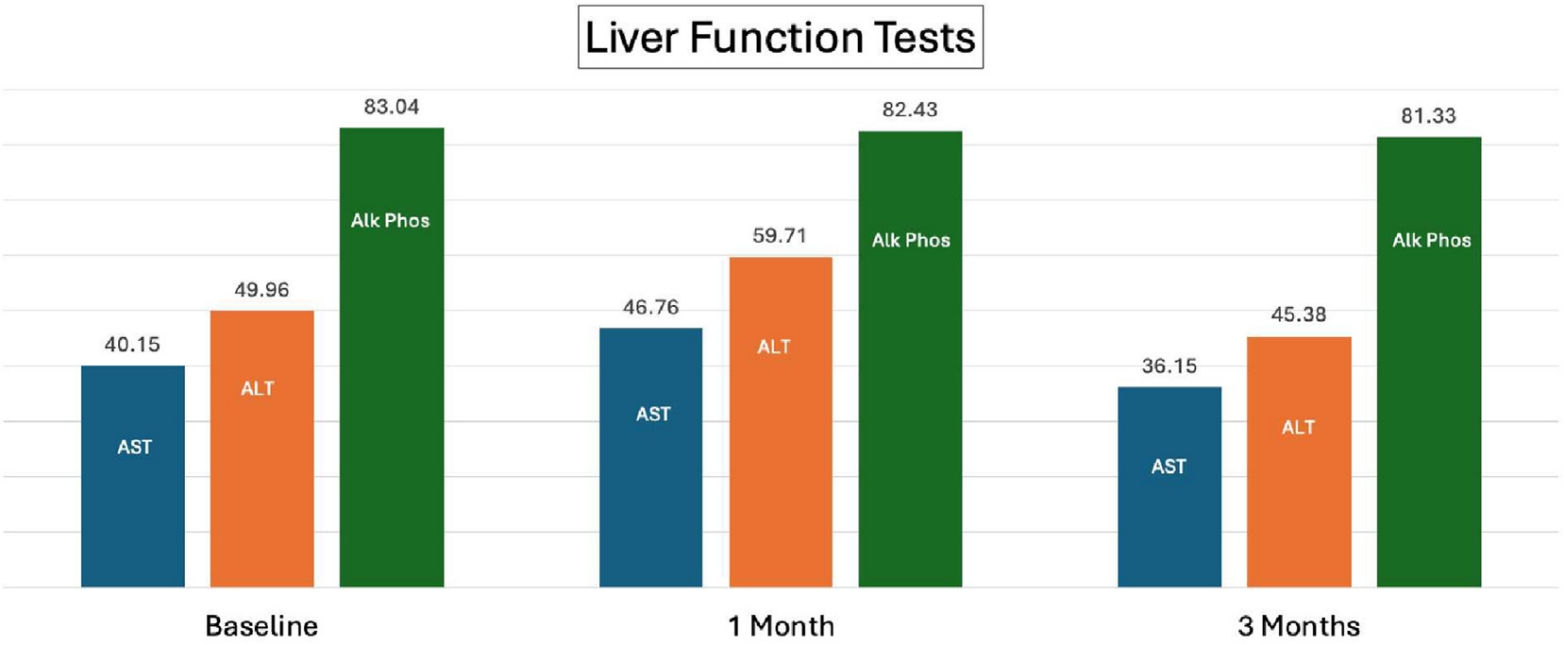
Mean AST, ALT, and ALP at baseline prior to starting the medication (left-most bars) were 40.15 IU/L, 49.96 IU/L, and 83.04 IU/L, respectively. At the 1-month treatment mark, the mean AST, ALT, and ALP (middle bars) were 46.76 IU/L, 59.71 IU/L, and 82.43 IU/L, respectively. At the 3-month treatment mark the mean AST, ALT, and ALP (right-most bars) were 36.15 IU/L, 45.38 IU/L, 81.33 IU/L, respectively.

At the 1-month treatment mark, the mean AST, ALT, and ALP were 46.76 IU/L (range: 13–282 IU/L), 59.71 IU/L (range: 7–311 IU/L), and 82.43 IU/L (range: 45–213 IU/L), respectively. At the 3-month treatment mark, the mean AST, ALT, and ALP were 36.15 IU/L (range: 7–170 IU/L), 45.38 IU/L (range: 11–223 IU/L), and 81.33 IU/L (range: 35–168 IU/L), respectively. The mean BMIs at the 1-month and 3-month treatment marks were 35.58 kg/m^2^ (range: 24.2–55 kg/m^2^) and 35.79 kg/m^2^ (range: 24.4–62.8 kg/m^2^), respectfully. There were 21 (18.6%) reports of elevated LFTs during the first 3 months (Figure 2).

Eleven patients were hospitalized on 14 occasions during the first 3 months of treatment with resmetirom; none of these hospitalizations were attributed to resmetirom use. One patient was seen in the emergency department for diarrhea but was discharged after intravenous fluids and was not admitted to the hospital. Other causes of hospitalization included chest pain/arrythmia-6, urinary tract infection-2, seizure disorder-1, dental abscess-1, osteoarthritis/knee pain-1, C. diff infection-1, insect bite-1, anxiety attack-1. A single patient accounted for 3 of the chest pain admissions. No deaths were reported of any patient involved in our study during the first 3 months of treatment.

### Pharmacy Access and Insurance Coverage Results

Among the 137 patients prescribed resmetirom, 129 patients (94.2%) required insurance prior authorization. A total of 33 (24.1%) insurance prior authorization denials were seen, which required an appeal for insurance approval. Of those, 128 patients (93.4%) were ultimately approved. Appeals were denied due to unmet biopsy requirements, expired noninvasive fibrosis testing, or complete plan exclusions. On average, resmetirom was dispensed 22.7 days after being prescribed, with a delay of 12.5 days after insurance approval. Some patients experienced delays exceeding 3 weeks postapproval. Factors contributing to dispensing delays included prior authorization requirements, additional fibrosis testing mandates, and patient-specific financial barriers.

Financial assistance in the form of manufacturer copay assistance, nonprofit foundation grants, and manufacturer patient assistance programs were utilized for 86 patients (62.8%), helping to mitigate cost burdens. Despite insurance approvals, access challenges persisted, with 5 patients discontinuing treatment prematurely due to cost concerns when nonprofit foundation grants were unavailable or logistical issues with specialty pharmacy dispensing. Notably, resmetirom prescriptions were dispensed primarily through a limited specialty pharmacy network, requiring additional coordination between providers, insurers, and pharmacy staff.

## Discussion

This retrospective chart review provides insights into the tolerability and adverse event profile of resmetirom in patients with MASLD during the first 3 months of treatment. The study population, characterized by a high prevalence of obesity (78%), diabetes mellitus (54%), hypertension (64%), and hyperlipidemia (72%), reflects a cohort representative of those typically seen with MASH in recent literature.^6^ These data also highlight the difficulties our patients faced in obtaining access to a brand new medication in the current insurance environment in the US.

## Treatment Continuation and Adherence

The high continuation rates at 1 month (95%) and 3 months (79%) suggest that resmetirom is generally well-tolerated in this population. While a small proportion (8.8%) discontinued treatment by 3 months, this included patients lost to follow-up, those who independently discontinued therapy, and those who were discontinued by their providers due to adverse effects or other reasons such as opting for lifestyle modifications. This relatively low discontinuation rate is encouraging, but the duration of treatment for this chronic condition is unclear at the moment and financial barriers may prove to be significant in the future. In general, however, discontinuation due to poor tolerability did not appear to be a major factor.

## Adverse Events

The most common adverse effects reported were gastrointestinal in nature, including nausea, vomiting, diarrhea, and abdominal pain. These findings align with the known adverse event profile of resmetirom and may reflect its mechanism of action on thyroid hormone pathways, which influence gastrointestinal motility and metabolism.^7^ Notably, a small number of patients (3%) experienced rash or pruritus, while only one patient developed hepatotoxicity. As previously mentioned, this patient encountered a minor bump in his LFTs the first month by a 2-fold increase which dropped back to normal levels by the third month of treatment. This low incidence of hepatotoxicity is promising, considering the underlying liver disease in this population.

Importantly, there were no reports of dizziness, cholecystitis, or pancreatitis, which are potential concerns in patients treated with lipid-modulating agents albeit these may become apparent with longer duration of treatment. Hospitalizations during the study period were generally not attributed to resmetirom although one patient was admitted with dehydration after diarrhea which may have been precipitated by the medication. However, 21 patients experienced minor elevated LFTs, highlighting the need for continued monitoring, especially in patients with advanced fibrosis or other risk factors for liver decompensation.

## Biochemical and Anthropometric Changes

At 3 months, mean AST and ALT levels improved compared to baseline, suggesting potential biochemical benefits of resmetirom in MASLD management. ALP levels remained stable, further supporting the safety profile. However, as expected from the published clinical trials, no significant BMI changes were observed during the study period.

### Insurance-Related Factors

Our findings highlight significant barriers to accessing resmetirom for MASH patients, despite FDA approval. Despite high insurance approval rates, nearly a quarter of patients required appeals, and dispensing delays were common. The average time from resmetirom being prescribed to being approved was about 11 days, potentially impacting early treatment initiation and adherence. Our institution has a specialty pharmacy with dedicated personnel who processed all these prescriptions and we therefore believe that delays were minimized. However, this may not be the case elsewhere. These delays were largely attributed to prior authorization requirements, additional invasive fibrosis testing mandates, insurance plan exclusions, and financial constraints. In the cases of complete plan exclusions on insurance plans, manufacturer patient assistance was attempted, which required a laborious approval process and uncertainty in continued access to the medication leading to potential premature discontinuation. While financial assistance programs helped offset costs for some patients, affordability remained a challenge when nonprofit foundation grants were unavailable, leading to a delay in medication initiation. Given the progressive nature of MASH, timely access to treatment is critical in slowing disease progression and improving patient outcomes.

Even with insurance approval and financial assistance, limited specialty pharmacy access introduced additional complexity, requiring extensive coordination between prescribers, insurers, and pharmacists causing an additional delay of 12.5 days. Overall, future efforts should focus on streamlining insurance approval processes, reducing unnecessary fibrosis testing requirements, and expanding specialty pharmacy networks to expedite medication dispensing. Additionally, increasing financial support options and advocating for broader coverage policies could enhance patient access. Addressing these barriers is crucial to optimizing resmetirom utilization, ensuring timely treatment access, and improving adherence.

### Clinical Implications and Limitations

These findings suggest that resmetirom has a favorable tolerability profile in patients with MASLD, with a high rate of treatment adherence and no unexpected adverse effects during the first 3 months. The observed biochemical improvements are encouraging and warrant further investigation in long-term studies to assess the drug's efficacy in reducing fibrosis and improving clinical outcomes. Previous literature has shown patients can achieve a 50%–64% relative reduction in hepatic fat.^8^ However, several limitations should be noted. The retrospective nature of this study limits causal inference and introduces potential biases, including incomplete documentation and variability in follow-up practices. The relatively short duration of follow-up limits the ability to assess long-term outcomes, and the absence of a control group precludes direct comparison of adverse events to standard care or placebo. Lastly, the small sample size and predominance of Caucasian patients may limit the generalizability of the findings to broader, more diverse populations.

## Conclusion

MASH is the most common liver disorder worldwide.^2^ This real-world study highlights the process that was used for identifying suitable patients for the first approved pharmacological agent for MASLD and the difficulties faced regarding access and insurance coverage. It also demonstrates that resmetirom in the real-world is a safe and well-tolerated option for patients with MASLD for at least the first 3 months of treatment.
